# n-3 Polyunsaturated Fatty Acids and Their Derivates Reduce Neuroinflammation during Aging

**DOI:** 10.3390/nu12030647

**Published:** 2020-02-28

**Authors:** Corinne Joffre, Anne-Laure Dinel, Mathilde Chataigner, Véronique Pallet, Sophie Layé

**Affiliations:** 1 Université de Bordeaux, INRAE, Bordeaux INP, NutriNeuro, 146 rue Léo Saignat, 33076 Bordeaux, France; mathilde@abyss-ingredients.com (M.C.); veronique.pallet@enscbp.fr (V.P.); sophie.laye@inrae.fr (S.L.); 2 NutriBrain Research and Technology Transfer, NutriNeuro, 146 rue Léo Saignat, 33076 Bordeaux, France; 3 Abyss Ingredients, 56850 Caudan, France

**Keywords:** aging, cognitive decline, n-3 polyunsaturated fatty acids, specialized pro-resolving mediators, resolution of inflammation, neuroinflammation

## Abstract

Aging is associated to cognitive decline, which can lead to loss of life quality, personal suffering, and ultimately neurodegenerative diseases. Neuroinflammation is one of the mechanisms explaining the loss of cognitive functions. Indeed, aging is associated to the activation of inflammatory signaling pathways, which can be targeted by specific nutrients with anti-inflammatory effects. Dietary n-3 polyunsaturated fatty acids (PUFAs) are particularly attractive as they are present in the brain, possess immunomodulatory properties, and are precursors of lipid derivates named specialized pro-resolving mediators (SPM). SPMs are crucially involved in the resolution of inflammation that is modified during aging, resulting in chronic inflammation. In this review, we first examine the effect of aging on neuroinflammation and then evaluate the potential beneficial effect of n-3 PUFA as precursors of bioactive derivates, particularly during aging, on the resolution of inflammation. Lastly, we highlight evidence supporting a role of n-3 PUFA during aging.

## 1. Introduction

Aging is a world concern as the elderly population tripled from 4% to 13% in the last century and is expected to grow sharply to reach 20% of the population in 2025 and 33% in 2050 [1]. Aging is associated to cognitive decline for 15–20% of the elderly >65 [2,3,4]. These cognitive alterations can lead to age-related disease such as neurodegenerative diseases. Alzheimer’s disease is the predominant one, affecting 24 million people in the world [5]. Thus, healthy aging constitutes a real economic challenge of the 21st century for the nations. The mechanisms explaining this process are still not fully elucidated, but neuroinflammation seems largely involved. Then, strategies to reduce and resolve neuroinflammation in a time-limited manner are encouraged. Recent studies suggest that nutrition, particularly fish oil, has promising anti-inflammatory effects. Fish oil contains n-3 long chain polyunsaturated fatty acids (LC-PUFAs), which are precursors of bioactive lipids called specialized pro-resolving mediators (SPMs) that largely contribute to this beneficial effect. Here, we review the effect of aging on neuroinflammation, in particular microglia activity and cognitive decline, and how n-3 LC-PUFAs and their derivates impact neuroinflammation, especially during aging. We discuss that nutrition, an environmental factor to which individuals are exposed throughout life, plays a key role to prevent or delay neuroinflammation during aging.

## 2. Aging and Neuroinflammation

Brain aging is associated to a chronic low-grade inflammation in the central nervous system (CNS) [6]. Microglial cells are the resident innate immune cells of the CNS and are involved in various physiological and pathophysiological functions [7,8]. These cells initiate the immune response when they recognize damage- (DAMPs) and pathogen-associated molecular patterns (PAMPs) thanks to their various pattern recognition receptors (PRRs), including toll-like receptors (TLRs) and nucleotide oligomerization domain (NOD)-like receptors [9]. They are strictly regulated by signals from the CNS [10] and with aging, they change their morphology, reduce their arborization, and decrease their mobility in human, non-human primates and rodents and then become senescent [11,12,13,14,15,16,17,18]. Indeed, aged microglia are “primed”, and are characterized by increased production of inflammatory markers, at baseline and in response to an immune stimulus, and by a decreased capacity to return to homeostasis [19,20,21]. Aged microglia also fail to degrade myelin fragments, resulting in the accumulation of lipofuscin granules, markers of microglial aging [22,23,24]. Thus, during aging, microglial functions change, resulting in increased immune age-related responses, driving the development of cognitive deficits, impaired synaptic plasticity and the progression of neurodegenerative pathologies [25,26]. These changes are mainly the result of age-induced defective mechanisms driving the inflammatory response [21,27].

During aging, under the basal condition, there is an increase in the expression of blood and brain levels of pro-inflammatory cytokines, including tumor necrosis factor α (TNF-α), interleukin-6 (IL-6), interleukin-1β (IL-1β), and interleukin-18 (IL-18), and a decrease in the expression of anti-inflammatory factors such as interleukin-10 (IL-10), interleukin-4 (IL-4), or brain derived neurotrophic factor (BDNF) [28,29]. Compared with young mice, aged mice have a higher expression of IL-6 in the hippocampus, cortex, and cerebellum [30,31], and a lower expression of IL-10 [32]. In aged microglial cells, there is a constant production of nuclear factor-kappa B (NFκB), a transcription factor involved in the activation of inflammatory pathways, leading to increased production of IL-6 [33]. Aged microglial cells from rodents produce more IL-1β and IL-6 than young ones [22,34,35,36]. Moreover, the serum level of IL-6 in elderly has been linked to the incidence of deficits in mobility and walking speed [37,38,39]. Markers of microglial activation are also increased during aging: major histocompatibility complex II (MHC II) [40,41], CD68 [42,43], caspase-1, as well as CD11b [44]. Indeed, in elderly without neurological pathologies, MHC II expression is related to increased brain IL-1β expression [45]. In the same way, *ex vivo* and *in situ* studies have shown that microglial cells of aged rats and mice display, compared with those of younger animals, a greater expression of MHC II, CD11b, and CD68—all markers of microglial cells’ activation [42,43]. The number of microglial cells expressing MHC II also increases with age in nonhuman primates [15] and in rats [18]. In the hippocampus, the number of microglial cells increases by 20% in aged mice compared with young adults [46].

The loss of homeostatic functions of microglia is a hallmark of unhealthy brain aging and neurodegenerative disorders [47]. Interestingly, recent studies using high-dimensional single-cell mapping or single cell RNAseq revealed that molecular signatures of microglia is altered with aging with some similar genes in rodents and humans [48,49,50]. The identification of aged-microglia subtypes allow to identify specific markers associated to unhealthy aging. Recent data pinpoint that mutations in triggering receptor expressed on myeloid cells 2 (Trem2) and colony stimulating factor 1 receptor (Csf1r) in microglia are responsible of neurodegenerative diseases, reinforcing the essential role of microglia in healthy aging. In elderly, the soluble form of Trem2 in the cerebrospinal fluid was associated to attenuated cognitive decline [51].

The increase in cytokine production in the blood and brain has been associated to age-related cognitive decline. IL-6 levels in the plasma of elderly have been positively correlated to cognitive decline, in particular to loss of speed of information processing [52,53,54]. This is in agreement with the fact that IL-6-deficient mice are protected from age-related decline of their cognitive performance following a bacterial endotoxin infection as compared with wild-type mice [55,56]. These mice also have less pro-inflammatory cytokines in the hippocampus. Moreover, in aged rodents, it is hippocampal IL-1β that is associated to impairment of learning and memory [57,58,59,60]. Pharmacological inhibition of IL-1β as well as its converting enzyme (ICE), which is essential for the release of IL-1β, has allowed to reduce memory impaiments induced by infection or stress in aged mice [61,62] and has improved the performance of aged rats [63]. Other studies have shown an increased expression of the NOD-like receptor protein 3 (NLRP3) in the hippocampus of aged mice, which regulates caspase-1 activation, and thus the maturation and secretion of IL-1β and IL-18 [64,65,66]. This NLRP3 activation by DAMPs as well as the production of reactive oxygen species (ROS) have been associated to age-related cognitive decline and neuropathological changes [67,68,69].

All these studies reveal that inflammation during aging characterized by microglial activation and pro-inflammatory cytokine production is partly responsible for age-related cognitive decline. Hence, reducing this low grade inflammation constitutes a good strategy to prevent age-related cognitive decline and the development of neurodegenerative pathologies.

## 3. N-3 PUFAs as Precursors of Lipid Mediators Involved in the Resolution of Inflammation

In the brain, the main n-3 LC-PUFA is docosahexaenoic acid (DHA), which represents 12–14% of total fatty acids in the brain [70,71,72,73,74,75] and has key-regulator functions in inflammation. Eicosapentaenoic acid (EPA) is the other n-3 LC-PUFAs of great importance, despite its low level in the brain because of its beta-oxidation [76]. Indeed, it is a precursor of many bioactive derivatives. N-3 LC-PUFAs can be synthesized from n-3 PUFA precursor alpha-linolenic acid (ALA), but the conversion rate is very low in humans [77,78] and becomes less efficient with aging [79,80]. Then, it is recommended to consume fish, which is the main dietary source of n-3 LC-PUFAs [80]. The absence of n-3 LC-PUFA consumption and/or a defect in their metabolism is responsible for increased neuroinflammation, leading to neurological disorders [81]. Indeed, numerous reviews have reported the powerful anti-inflammatory properties of n-3 LC-PUFAs [82,83,84,85,86].

Several mechanisms have been proposed to explain the immunomodulatory properties of n-3 LC-PUFAs. One of the most attractive is the synthesis of bioactive lipid mediators or oxylipins. These oxylipins are synthesized sequentially: first, those involved in the regulation of inflammation such as the eicosanoids (prostaglandins, leukotrienes, thromboxane), and then those involved in the resolution of inflammation called SPMs (resolvins, protectins, maresins). SPMs have both anti-inflammatory and pro-resolutive properties without immune suppression and induce a return to homeostasis [87,88,89,90]. They actively coordinate and finely tune the inflammatory response. They down-regulate the pro-inflammatory cytokines and up-regulate the anti-inflammatory cytokines, promote the phagocytosis of cellular debris and dead cells without immune suppression, reduce the concentration, and compete with pro-inflammatory oxylipins derived from n-6 PUFAs. Then, they underlie most of the beneficial effects attributed to their precursors [84,91,92,93]. Several enzymes are responsible for their synthesis: phospholipases A2 (PLA2s) for the release of fatty acids from the membranes, as well as cyclooxygenase (COX)-2, lipoxygenase (LOX), and cytochrome P450 monoxygenases (CYP450) [94]. They convert DHA and EPA into bioactive lipid mediators. In human serum, DHA- and EPA-derivates represent 30.7% and 25.9% of the identified SPMs, respectively [95,96]. These enzymes are expressed in the brain [97,98,99,100]. Following an inflammatory stimulus such as lipopolysaccharide (LPS), COX-2 is rapidly expressed in the hippocampus [100,101]. It was shown that COX-2 inhibition delays resolution of acute inflammation [102]. 15-LOX and 5-LOX are the most abundant LOX in the brain [97]. 15-LOX is both neurotoxic owing to the oxidative stress it generates [103] and neuroprotective owing to the SPMs it synthesizes [104,105]. Indeed, the impairment of 15-LOX activity (by gene deletion or pharmacological inhibition) reduces the SPM production in the brain and is associated to cognitive alterations [97]. CYP450 produces anti-inflammatory n-6 derived epoxides [106,107,108,109]. These enzymes have also been identified in brain cells such as microglia, astrocytes, oligodendrocytes, and neurons [110,111,112,113].

### 3.1. DHA-derived SPMs 

**Figure 1:**
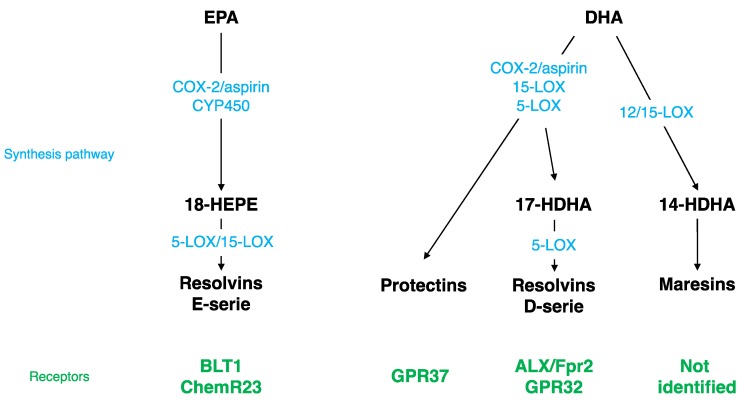
Specialized Pro-resolving Mediator (SPM) synthesis pathway and receptors. EPA, eicosapentaenoic acid; DHA, docosahexaenoic acid; LOX, lipoxygenase; COX, cyclooxygenase; ALX/Fpr2, lipoxin A4 receptor/formyl peptide receptor 2; GPR32, G protein coupling receptor 32; BLT1, leukotriene B4 receptor; HDHA, monohydroxy DHA; CYP450, cytochrome P450 monoxygenases.

Different SPMs can be synthesized from DHA (Figure 1): monohydroxy DHA (17-HDHA) by acetylated COX-2, CYP450, and 15-LOX [114,115] and resolvin D1 (RvD1) *via* the production of 17-HDHA by 5-LOX [116,117]. These bioactive derivates have been mostly described at the periphery, but have also been detected in the brain. RvD1 was measured in mouse brain following cerebral ischemia [118]. Its level is modulated by a DHA intravenous injection [119] and during inflammation; it decreases at the beginning and then increases during the resolution phase [120]. RvD1 acts at picomolar range, but exerts its biological effects at nanomolar range [117,121]. The receptor of RvD1 is lipoxin A4 receptor/formyl peptide receptor 2 (ALX/FPR2) in rodents and G protein coupling receptor 32 (GPR32) in humans [116]. Several brain structures express ALX/FPR2: brainstem, spinal cord, hypothalamus, cortex, hippocampus, cerebellum, and striatum [122]. At the cellular level, these receptors are expressed in microglial cells [123], neurons [122,124], and astrocytes [125,126]. *Via* these receptors, RvD1 regulates micro-RNAs (miRNAs), which play a key role in modulating the expression of target genes such as inflammatory genes [123,125,127,128,129].

Other SPMs are derived from DHA: di-hydroxy-DHA termed protectin D1 (PD1) or neuroprotectin D1 (NPD1) when produced in the CNS by 5- and 15-LOX [130,131,132,133], and maresin 1-2 (MaR1-2) by 12/15-LOX [114,115,134]. NPD1, MaR1, and its precursor 14-HDHA were measured in the hippocampus [135]. The level of NPD1 and MaR1 decreases in the hippocampus of Alzheimer’s disease patients [136,137] and the level of NPD1 greatly increases following brain ischemia or acute central LPS injection [118,135]. NPD1 receptor has been identified only at the periphery in macrophages, but not in microglia [138], whereas the MaR1 receptor has not been identified yet [136]. NPD1 regulates NFκB, and then consequently pro-inflammatory gene expression [118,139,140]. MaR1 decreases pro-inflammatory signaling cascades and influences macrophages towards an M2 repair phenotype after cerebral ischemia or spinal cord injury [141,142,143].

### 3.2. EPA-derived SPMs 

EPA is converted by acetylated COX-2 or CYP450 into 18R-HEPE, which is then metabolized into resolvins E1, E2, and E3 by 5- or 15-LOX (Figure 1) [114,144,145]. These derivates have been detected in the hippocampus [135,146,147]. RvE1 induces a decrease in LPS-induced pro-inflammatory cytokines’ (TNF-α, IL-6, IL-1β) gene expression in microglial cells by inhibiting the NFκB signaling pathway [123]. The receptors of RvE1 are a G protein coupling receptor, ChemR23, or chemokine like receptor 1 (CMKLR1) [144] and a leukotriene B4 receptor (BLT1) [148]. ChemR23 has been identified in the prefrontal cortex, hippocampus, and brainstem [149]. These receptors are also expressed in microglial cells [123,150], neurons [122,124], and astrocytes [126].

## 4. Role of Lipid Mediators in the Resolution of Inflammation

A large number of studies support the beneficial role of n-3 LC PUFAs in inflammation in human and animal models of acute and chronic inflammation, including in the brain (for recent reviews, see [82,83]). Here, we will review the biological roles at the brain level of RvD1 and RvE1, two distinct lipid mediators generated from the n-3 LC-PUFAs DHA and EPA, known for their powerful anti-inflammatory and pro-resolutive properties.

### 4.1. In Humans 

The effect of RvD1 was mainly studied in Alzheimer’s and Parkinson’s patients (Table 1). In patients with dementia, the levels of RvD1 in cerebrospinal fluid are positively associated with the improvement of cognitive functions [126]. RvD1 promotes Aβ phagocytosis by macrophages isolated from Alzheimer’s patients, reducing the amyloid load [151,152]. Moreover, as cited in Krashia et al., endogenous RvD1 is decreased in patients diagnosed with early-Parkinson’s disease [153]. As a result, the decrease of RvD1 levels in Alzheimer’s and Parkinson’s disease patient’s brain could contribute to the disease development and progression. Conversely, an increased anti-inflammatory RvD1 activity has been reported in maniac and depressive patients, suggesting that RvD1 may serve to improve inflammatory imbalance [154].

The effect of RvE1 was reported in patients at the periphery (Table 1) [155,156,157], but not at the brain level. Hence, more studies are needed to develop this area. 

### 4.2. In Animals 

Several studies report that, in rodent models of inflammation, RvD1 and RvE1 display anti-inflammatory activities in the CNS (Table 2). Indeed, RvD1 reduces the activation of NFκB and the expression of pro-inflammatory factors such as IL-1β, IL-6, TNF-α, and iNOS in rats with hemorrhagic shock or in streptozotocin (STZ)-induced diabetic retinopathy [158,159]. RvD1 attenuates neuroinflammation through ALX-FPR2 receptor *via* miRNA in a neonatal hypoxia-ischemia rat pup model or in a remote damage model [125,160]. Moreover, RvD1 induces the polarization of macrophages and microglia toward an M2 phagocytic phenotype [161,162,163]. Chronic and early RvD1 administration in a rat model of Parkinson’s disease prevents central and peripheral inflammation, as well as neuronal dysfunction and motor deficits [153]. In addition, the precursors of RvD1, 17R-HDHA and 17S-HDHA, reduce the production of pro-inflammatory cytokines in the spinal cord and in the hippocampus [135,164]. 

RvE1 reduces the expression of pro-inflammatory cytokines IL-1β and IL-6 in the prefrontal cortex and decreases the neuropathological features of Aβ pathology in a murine model of Alzheimer’s disease [165]. Furthermore, repeated RvE1 administration moderates the activation of microglia by promoting ramified microglia following traumatic brain injury or peripheral brain injury [166].

The effect of RvD1 on neuroinflammation is associated to effects on cognition. Indeed, RvD1 prevents cognitive deficits. In a rodent model of systemic inflammation or traumatic brain injury, an intraperitoneal administration of 17R-RvD1 prevents cognitive decline [166,167]. Of note, higher levels of brain RvD1 in Fat-1 mice, owing to higher brain n-3 LC-PUFAs induced by genetic means, are linked to less cognitive deficits, a reduction in microglial activation, and in pro-inflammatory status following brain ischemia [168,169]. Conversely, lower levels of brain RvD1, owing to 15-LOX inhibition, are related to alterations in working memory and synaptic plasticity in rats [97].

RvD and RvE have been reported to prevent emotional behavior alterations in rodent models of mood disorders in the review of Furuyashiki et al. [170]. These SPMs have positive effects in LPS-induced or chronic stress-induced or post-myocardial infarct depression [164,171,172,173,174,175,176].

### 4.3. In Vitro 

The effects of RvD1 and RvE1 were tested on different brain cells, highlighting their pro-resolutive properties (Table 3). In microglial cells, RvD1 enhances the effect of the anti-inflammatory cytokines IL-4, Arg1, and Ym1 and reduces the activation of microglia by decreasing CD11b expression, leading to a more anti-inflammatory phenotype of microglia [163,177,178]. Moreover, RvD1 reduces LPS-induced pro-inflammatory cytokine (TNF-α, IL-6, and IL-1β) gene expression in microglial BV2 cells by regulating miRNA expression [123]. It was also reported that RvD1 down-regulates Aβ-induced inflammation in human microglia [136]. RvD2 decreases the expression of toll like receptor 4 (TLR4, the receptor of LPS) following LPS treatment, and consequently its downstream signaling pathway NFκB [179]. RvE1 also reduces microglial activation and pro-inflammatory cytokine release in microglial cells [123,177]. In astrocytes, RvD1 decreases LPS-induced TNF-α production [164]. In neurons from spinal nods, RvD1 increases neurite outgrowth [180]. In PC12 neural cells, used as an *in vitro* model of Parkinson’s disease, RvD1 reduces TNF-α and IL-6 mRNA expression [181]. The anti-inflammatory properties of RvD1 were also tested in macrophage cells. RvD1 reduces the expression of pro-inflammatory markers (cytokines, PGE2) and increases anti-inflammatory cytokine IL-10 in murine macrophages stimulated by LPS [182]. RvD1 polarizes primary human macrophages toward a pro-resolutive phenotype through GPR32 receptor [183].

## 5. Defects in Lipid Metabolism and Lipid Mediator Production during Aging 

During aging, brain levels of n-3 LC-PUFAs decrease, although all brain structures are not affected in the same way [30,32,70,184]. This reduction was observed in human [185,186], especially in the cortex, the hippocampus, and the cerebellum [73,187,188,189], and in rodents [30,32,190,191], in particular in the hippocampus [191] and the cortex [73], which are key structures in mnesic processes. This decrease is mainly because of changes in lipid metabolism: alteration in the intestinal absorption of essential fatty acids [192,193,194]; impairment in the enzymes of phospholipid synthesis [195]; reduced conversion rates of the precursors into LC-PUFAs owing to reduced activity of the enzymes involved in their synthesis, in particular of Δ6 desaturase [186,196,197]; and modifications in the expression of the genes implicated in the metabolism of PUFAs. Indeed, single nucleotide polymorphisms (SNPs) in desaturase genes *FADS1* (Δ5 desaturase), *FADS2* (Δ6 desaturase), as well as *ELOVL2* (elongase 2) are related to higher ALA and lower EPA plasma phospholipid levels with age, suggesting different rates of conversion [198]. Moreover, another possible reason of the decrease of n-3 LC-PUFAs in the membranes is their high propensity to oxidation to generate peroxidation products such as malonaldehyde (MDA), 4-hydroxy-2-nonenal (4-HNE), or 4-hydroxy-2-hexenal (4-HHE). Indeed, levels of MDA and 4-HNE are increased in aged brain of humans and rodents [199,200].

**Figure 2:**
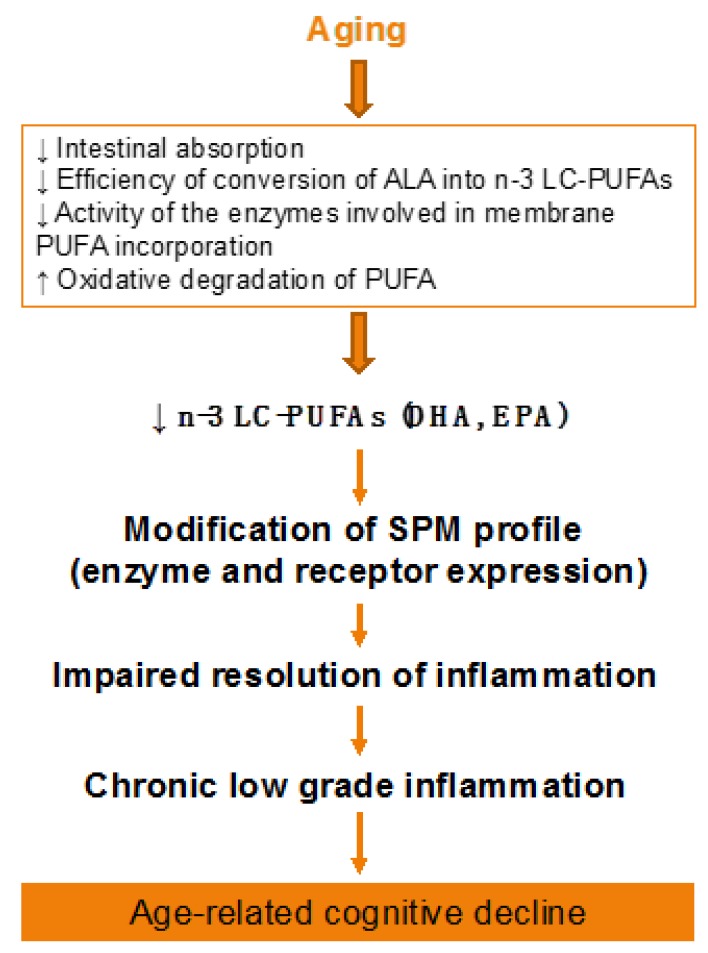
**Effect of aging on lipid metabolism**. ALA, alpha-linolenic acid; PUFA, polyunsaturated fatty acid; SPM, specialized pro-resolving mediator.

Aging-associated DHA metabolism disturbance could participate in cognitive decline (Figure 2). This has been demonstrated both in humans and animals. In elderly, decreased n-3 PUFA consumption associated to reduced erythrocyte DHA levels are inversely correlated with age-related cognitive decline [201,202,203]. In rats, a low-DHA dietary supply for one or more generations is related to alterations in cognitive function [204,205,206]. In addition, we showed in aged mice that an n-3 PUFA deficient diet impairs memory as well as neuroinflammation and synaptic plasticity [32,207,208,209,210]. Furthermore, the decrease in brain DHA content induced by a n-3 PUFA deficient diet increases vulnerability to inflammation, which trigger both synaptic and memory alteration [211,212]. On the contrary, a two-month n-3 LC-PUFA supplementation in aged mice (between 20 and 22 months old) reverses age-induced spatial memory deficits [30].

Age-related alteration of n-3 PUFA metabolism contributes to reducing the n-3 LC-PUFA content in brain phospholipids. As n-3 LC-PUFAs are precursors of bioactive mediators involved in the resolution of inflammation, it may have consequences on SPM profile and production. Indeed, it was recently shown that blood oxylipin profile is altered in 45–64-year-old healthy men and women *versus* 19–28-year-old young people [213,214]. Moreover, Gangemi et al. (2005) demonstrated that aging is associated to a decrease in urinary LxA4/leukotriene, a ratio of anti-inflammatory/pro-inflammatory mediators synthesized from arachidonc acid and considered as an index of the endogenous anti-inflammatory potential [215]. Moreover, LxA4 is significantly lower in cerebrospinal fluid (CSF) of humans with Alzheimer’s disease as compared with humans with mild cognitive impairment or subjective cognitive impairment, with a positive correlation between CSF LxA4 and cognitive performance [126].

In animals, oxylipin profile modification was also reported with aging. Aged rodent brains display higher levels of TxB2, 6-keto-PGF1α, and PD1-like metabolites [214]. In a model of senescence-accelerated prone mice (SAMP8), the cortex contains higher levels of PGE2, TxB2, and 9,10-DiHOME and lower levels of 20-HETE and DHA-derived mediators (11-, 14-, and 20-HDoHE) [214]. However, when compared with same age senescent-accelerated mouse resistant 1 (SAMR1) mice, SAMP8 mice do not exhibit any difference in LXA4 or RvD1 levels, despite a greater degree of inflammation in SAMP8 mice [216]. Moreover, aged BalbC mice display higher levels of pro-inflammatory LTB4 and PGs, but lower anti-inflammatory RvD1 and MaR1 in peritoneal macrophages compared with young mice [217]. 

The modifications of oxylipin profile are linked to changes in the expression of the enzymes involved in oxylipin formation. In humans, the expression of PLA2 and LOX increases with aging in *post-mortem* brain [214]. Similar results were obtained in 70-year-old *versus* 41-year-old patients concerning PLA2 and CYP [214]. In Alzheimer’s disease patients, 15-LOX level is also increased in the hippocampus [126].

In animals, the expression of 5-LOX is increased with aging [214] whereas the expression of 12-LOX is decreased in nine-month-old SAMP8 mice [216].

The changes in oxylipin profile may have compensatory consequences on their receptors. Indeed, in humans, ALX/FPR2 and ChemR23 levels are higher in the hippocampus of Alzheimer’s disease patients as compared with controls [126]. A similar result was obtained for ALX/FPR2 in SAMP8, despite that its level is similar to the SAMR1 controls [216].

All these results suggest an altered resolution of inflammation during aging that may contribute to the age-related cognitive decline, as high inflammation is associated to altered cognition.

## 6. Evidence Supporting a Role of Dietary n-3 PUFAs during Aging

Bioactive nutrients such as n-3 PUFAs constitute an interesting potential way to prevent or delay neuroinflammation that occurs during aging. Here, we will focus on dietary n-3 PUFAs because they modify the levels of brain n-3 LC-PUFAs [83,84,218] that are both anti-inflammatory and pro-resolutive and prevent cognitive decline associated to aging.

Evidence in humans (Table 4) and animals (Table 5) supports a powerful role of n-3 LC-PUFAs in the regulation of both inflammatory pathways, and *in fine*, in the resolution of inflammation, including in the brain (recently reviewed in [83]). Here, we will focus on dietary supplementation using n-3 LC PUFAs during aging. Barberger-Gateau highlighted in elderly that the more they eat n-3 PUFAs, the less they are subjected to cognitive decline [219]. Similarly, Tan et al. showed in the Framingham Study participants that lower erythrocyte DHA levels are related to cognitive impairment [220]. Moreover, in a prospective observational study, baseline dietary DHA intake levels at 70 years old are positively correlated with a better declarative memory test performance at the age of 75 in a healthy population [221]. Dietary supplementation with n-3 PUFAs conducted in humans has been motivated by observational studies showing the link between dietary consumption of DHA and improved cognitive function and/or reduced cognitive decline in the elderly. Indeed, fish oil consumption, leading to increased levels of DHA in erythrocytes, has been associated with better cognitive performance in elderly [222] and with a lower risk of developing neurological disorders [223,224,225]. DHA dietary supply is associated to better performance and speed in a verbal learning test in a cohort of 45–70-year-old healthy individuals [226] and to improved mini mental state examination (MMSE) scores, used to evaluate cognitive functions and memory abilities, in a cohort of elderly of 75-year-olds [227]. Yurko-Mauro et al. have shown in a systematic meta-analysis that DHA intake improves episodic, working and semantic memories [228]. More recently, McNamara et al. have revealed that fish oil consumption decreases self-reported inefficiencies in everyday functioning as well as improves cognition in elderly with cognitive complaints [229]. Moreover, circulating n-3 PUFAs (including DHA) have been negatively associated to the level of cytokines [230,231,232].

Beneficial effects of n-3 LC-PUFAs have also been found in animals. Administration of a DHA/EPA diet to aged mice protects against neuroinflammation and cognitive impairment [30] and improves spatial cognition and learning ability and memory [233,234]. Interventional studies in aged rodents have demonstrated that the ingestion of a fish oil-enriched diet decreases the *ex vivo* production of IL-1β, TNF-α, and IL-6 by monocytes and macrophages [235,236,237]. Moreover, circulating concentrations of IL-1β, TNF-α, and IL-6 following LPS injections are lower in rats and mice fed a fish oil-enriched diet [238,239,240]. Furthermore, age-related brain expression of pro-inflammatory cytokines in rodents is reduced with high levels of DHA [241].

In addition, it is possible to modulate oxylipin profile *via* dietary interventions. Indeed, as reviewed by Caligiuri et al. in human blood, the oxylipin profile is changed towards a less inflammatory profile after n-3 LC-PUFA consumption [214]. We found that in mice treated with LPS, a brain n-3 LC-PUFA increase by dietary supplementation promotes the synthesis of n-3 PUFA derived SPMs and decreases n-6 PUFA-derived SPMs displaying an anti-inflammatory profile [100]. Moreover, increased plasmatic pro-inflammatory oxylipins in elderly is reversed by dietary n-3 PUFA (alpha-linolenic acid, the precursor of n-3 LC-PUFAs) [213]. The OmegAD study revealed that Alzheimer’s disease patients treated with n-3 PUFAs preserve their RvD1 levels as compared with placebo-treated patients [242]. In aged rats, n-3 LC-PUFA supplementation increases DHA-derived oxylipins in the cortex and improves the reference memory-related ability learning [243].

The modification of SPM levels in blood and brain cells of aged human and rodents is accompanied by some modification of the expression of their enzymes involved in their synthesis. 15-LOX mRNA expression increases in n-3 LC-PUFA supplemented group and decreases in n-3 LC-PUFA deficient diet [100,244,245]. 15-LOX generates both 15-HETEs that inhibit NFκB [103] as well as RvD1 that contributes to the preservation of cognitive performance [97]. 

These results suggest that dietary habits may be essential regulators of oxylipin profile and reinforce the importance of the recommendation of n-3 PUFA rich diet. 

## 7. Conclusions

In conclusion, aging is characterized by low-grade neuroinflammation, in particular, activation of microglial cells and increase in the production of brain pro-inflammatory factors, such as cytokines. This neuroinflammation is associated with cognitive decline (15–20% of the >65-year-old elderly), which affects life quality and has a major economic and social impact. In this context, it is a priority to find strategies to delay the evolution towards neurodegenerative diseases. n-3 LC-PUFAs and their bioactive lipid derivates (SPMs) are promising as they reduce and resolve inflammation. SPMs are modulated by aging and dietary means reinforcing the importance of nutrition in the regulation of inflammation. Changes in dietary n-3 PUFA balance should have dramatic consequences in brain PUFA metabolism, and finally in the response to neuroinflammation particularly during aging. More studies are needed to confirm the role of SPMs in age-related changes, with this field being yet in emergence, and to investigate the interest to combine different oxylipins to potentiate their beneficial effects during aging. The clinical form (encapsulated SPMs or more stable-SPM analogues), the doses, and the way of administration should also be defined.

## Figures and Tables

**Table 1:** Role of lipid mediators in the resolution of inflammation in humans.

Ref.	Authors	Year	Subjects	Supplementation	Key Findings
[126]	Wang et al.	2015	AD, MCI, and SCI patients; 57–68 yrs	-	RvD1 levels in CSF correlate to MMSE scores
[151]	Famenini et al.	2017	MCI, SCI that are E3/E3 or E3/E4	1000 mg DHA + 1000 mg EPA/d for 35 months	RvD1 decreases the M1/M2 ratio in patients with ApoE E3/E3, improving Aβ phagocytosis
[152]	Mizwicki et al.	2013	Sporadic AD patients and controls	-	RvD1 rebalances inflammation to promote Aβ phagocytosis
[153]	Krashia et al.	2019	Early Parkinson’s disease patients		Decreased endogenous level of RvD1 correlates to increased levels of pro-inflammatory factors in CSF
[154]	Kok Kendirlioglu et al.	2019	Bipolar disorder-I patients	-	The increase in RvD1 during manic and depressive states improves inflammatory imbalance
[155]	Uno et al.	2016	Patients undergoing hepatobiliary resection	Oral supplementation of Oral Impact (Nestlé Health Science), 1000 kcal/d containing EPA and arginine for 5 consecutive days before the operation	Increased production of plasma RvE1 suppresses operation-induced acute inflammation
[156]	Hiram et al.	2015	Human pulmonary arteries	-	RvE1 resolves human arterial hyperreactivity via the resolution of inflammatory markers

AD: Alzheimer’s disease; ApoE: apolipoprotein E; CSF: cerebrospinal fluid; EPA: eicosapentaenoic acid; MCI: mild cognitive impairment; MMSE: mini-mental status examination; RvD1: resolvin D1; RvE1: resolvin E1; SCI: subjective cognitive impairment.

**Table 2:** Role of lipid mediators in the resolution of inflammation in animals.

Ref.	Authors	Year	Animals	Treatment	Key Findings
[97]	Shalini et al.	2018	Adult rat	Alox15 knock-down	Decrease in RvD1 levels in the prefrontal cortex associated to alteration in working memory performance
[125]	Bisicchia et al.	2018	Adult rats	Intraperitoneal injection of RvD1 (0.4 µg/kg) 3, 5, and 7 days after HCb lesion	RvD1 reduces glial activation and prevents neuronal death, promoting functional recovery
[135]	Orr et al.	2013	12 weeks C57BL/6J	Intracerebroventricular administration of 17S-HpDHA (1 µg) over 24 h *via* osmotic pump	17S-HpDHA attenuates hippocampus neuroinflammatory markers
[153]	Krashia et al.	2019	Syn rats (overexpressing the full-length human SNCA locus under the control of the endogenous human regulatory elements)	Intraperitoneal injection of RvD1 (0.2 µg/kg) twice a week for 8 weeks	RvD1 prevents microglial activation, and reduces CSF IFN-γ and MHC-II expression, and neuronal and motor deficits
[158]	Sordi et al.	2019	Hemorrhagic shock-induced rats	Intravenous injection of RvD1 (0.3 or 1 µg/kg)	Administration of RvD1 on resuscitation inhibits NFκB activation and reduces the expression of pro-inflammatory factors
[159]	Yin et al.	2017	STZ-induced diabetic retinopathy rats	Intravitreal administration of RvD1 (1000 ng/kg)	RvD1 inhibits the activation of the NLRP3 inflammasome and associated cytokine production
[160]	Liu et al.	2019	Hypoxic-ischemic induced 10-day old rat pups	Intraperitoneally injection of RvD1 (5 µg/kg) 1 h before hypoxia-ischemia	RvD1 administration reduces percent infarction area, microglia activation, and pro-inflammatory factor level
[161]	Rossi et al.	2015	Footpad-LPS injected rats	Intravitreal administration of RvD1 (10, 100, 1000 ng/kg)	RvD1 decreases the ocular damage reducing the presence of B and T lymphocytes, changing the expression of miRNA and the polarization of local macrophages and decreasing the local levels of ubiquitin-proteasome system
[162]	Titos et al.	2011	Peritoneal macrophages from C57BL/6J mice	10 nM RvD1	RvD1 polarizes macrophages toward a M2 phenotype and promotes macrophages phagocytosis
[164]	Abdelmoaty et al.	2013	Adult rats	Intrathecally administration of 17R-RvD1 (300 ng)	17R-RvD1 attenuated carrageenan-induced spinal TNF-α release
[165]	Kantarci et al.	2018	5xFAD female mice co-expressing human APP and PS1 with multiple FAD mutations	Intraperitoneally RvE1 injection (1.5 µg/kg) three times a week, for 2 months	RvE1 restores the expression of three SPMs and the cytokine levels in the prefrontal cortex
[166]	Harrison et al.	2015	TBI-induced C57BL/6 mice	Intraperitoneally RvE1 or 17R-RvD1 injection (100ng) for 7 consecutive days, beginning 3 days before TBI induction	RvE1 and 17R-RvD1 reduce microglial activation and promote microglial ramification. 17R-RvD1, but not RvE1 reduces cognitive deficits.
[167]	Terrando et al.	2013	Tibia-fracture induced C57BL6	Intraperitoneally 17R-RvD1 injection (100 ng) before surgery	17R-RvD1 reduces plasma IL-6 levels 6 h and 24 h after surgery
[168]	Delpech et al.	2015	LPS-treated Fat-1 mice	-	The increase in brain n-3 PUFA reduces LPS-induced pro-inflammatory cytokine production and subsequent spatial memory alteration
[169]	Luo et al.	2014	Transient cerebral ischemia Fat-1 mice	-	Suppression of NFκB activation, decrease in pro-inflammatory mediators, reduction in microglial activation, and increase in RvD1 level in hippocampus. Less severe hippocampal CA1 neuronal loss and cognitive deficits
[171]	Deyama et al.	2017	LPS-induced depression model Balb/c mice	Intracerebroventricular infusion of RvD1 (10 ng) and RvD2 (10 ng), 22 h after LPS challenge	Antidepressant effect of RvD1 and RvD2 through mTORC1 signaling pathway
[172]	Deyama et al.	2018	LPS-induced depression model Balb/c mice	Intracerebroventricular infusions of RvE1 (1 ng) or RvE2 (10 ng), 22h after LPS challenge	Antidepressant effect of RvE1 and RvE2 *via* ChemR23 in the prefrontal cortex and hippocampus
[173]	Deyama et al.	2018	LPS-induced depression model Balb/c mice	Intracerebroventricular infusions of RvE3 (10 or 100 ng), 22 h after LPS challenge	Antidepressant effect of RvE3
[174]	Klein et al.	2014	Fibromyalgia-like model Swiss mice	Intravenous administration RvD1, 17R-RvD1, or RvD2 (300 ng/mouse) 30 min after fibromyalgia induction and 4 days after, 30 min before behavioral evaluation	17R-RvD1 and RvD2 (but not RvD1) reduce painful and depressive symptoms
[175]	Gilbert et al.	2014	Myocardial infarction induced rats	n-3 PUFA rich diet for 10 days before myocardial infarction + RvD1 injection in the left ventricle the 10th day, 5 min before ischemia	RvD1 restores cardioprotection when added to the inhibitors of 15-lipoxygenase and of cycloxygnase-2
[176]	Ishikawa et al.	2017	Chronic unpredictable stress-induced depression model Balb/c mice	Intracerebroventricular RvD1 or RvD2 (10ng) infusion	RvD1 and RvD2 ameliorate depressive-like behavior

ChemR23: Chemerin Receptor 23; CSF: cerebrospinal fluid; HCb: hemicerebellectomy; HpDHA: hydroperoxyl-docosahexaenoic acid; LPS: lipopolysaccharides; MHCII: major histocompatibility complex II; mRORC1: mammalian target of rapamycin complex 1; NLRP3: NOD-like receptor family, pyrin domain containing 3; PUFA: polyunsaturated fatty acid; RvD: resolvin D; RvE: resolvin E; SPM: specialized pro-resolving mediators; STZ: streptozotocin; TBI: traumatic brain injury.

**Table 3:** Role of lipid mediators in the resolution of inflammation in vitro.

Ref.	Authors	Year	Cells	Treatment	Key Findings
[123]	Rey et al.	2016	BV-2 microglial cells	10 nM RvD1 or RvE1, 30 min before LPS treatment and during 24 h	RvD1 and RvE1 both decreased LPS-induced proinflammatory cytokines (TNF-α, IL-6, and IL-1β) gene expression *via* miRNA for RvD1 and NFκB pathway for RvE1
[136]	Zhu et al.	2016	Human CHME3 microglial cells	0–0.5 µM RvD1 for 1 h and 6 h	RvD1 down-regulates Aβ42-induced inflammation via the reduction in microglial activation
[163]	Li et al.	2014	BV-2 microglial cells	1, 10, or 100 nM RvD1 for 30 min before addition of 10 ng/mL murine IL-4	RvD1 enhances the IL-4-induced M2 polarization
[164]	Abdelmoaty et al.	2013	Rat primary astrocytes	500 nM 17-R-RvD1, 30 min before IFN-γ or LPS stimulation and during 24 h	17-R-RvD1 attenuates IFN-γ or LPS-induced TNF-α production
[177]	Xu et al.	2013	Primary microglial cells	1, 10, 100 ng/mL RvE1, 15 min before LPS treatment and during the 24 h LPS treatment	RvE1 suppresses LPS-induced microgliosis and prevents TNF-α release
[179]	Tian et al.	2015	Rat primary microglial cells	1.25, 2.5, 5, 10, 20 µM RvD2, 2 h before LPS treatment and during the 2 h LPS treatment	RvD2 reduces LPS-induced inflammatory markers (TNF-α, IL-6, IL-1β, IL-18, NO, TLR4, NFκB, IκB) and microglial activation markers (Iba1, CD11b)
[180]	Shevalye et al.	2015	Mouse primary neurons	50 nM RvD1 for 24 h	RvD1 increases neurite outgrouth
[181]	Xu et al.	2017	PC12 Parkinson’s disease model cells	50, 100, 200 nM RvD1, 2 h prior MPP+ treatment	RvD1 attenuates MPP+ upregulation of TNF-α and IL-6 mRNA expression via the inhibition of the activation of p38/ERK and NFκB signaling pathways
[182]	Benabdoun et al.	2019	Murine macrophage RAW 264.7	100, 200, 500 nM RvD1 for 72 h	RvD1 reduces LPS-induced PGE2 and TNF-α production, and increases IL-10 production
[183]	Schmid et al.	2016	Human primary macrophages	10 nM RvD1 for 48 h	RvD1 decreases IL-1β and IL-8 secretion and tends to reduce MCP-1 via the activation of GPR32

RvD1: resolvin D1; RvE1: resolvin E1; GPR32: G protein-coupled receptor 32; MPP+: 1-methyl-4-phenylpyridinium ion; PGE2: prostaglandin E2.

**Table 4:** Evidence supporting a role of dietary n-3 PUFAs during aging in humans.

Ref.	Authors	Year	Subjects	Supplementation	Key Findings
[219]	Barberger-Gateau	2009	Three cities cohort participants (75.9 years old)	Mediterranean diet for 5 years	Higher Mediterranean diet adherence associated to better cognitive performance assessed
[220]	Tan et al.	2012	Dementia-free Framingham cohort participants (67 years old)	Dietary habits	Lower red blood cell DHA level associated to lower scores on tests of visual memory, executive function, and abstract thinking
[221]	Titova et al.	2013	PIVUS cohort participants (70 years old)	Dietary habits for 5 years	A 7-day dietary intake of EPA and DHA positively associated with increased global cognitive performance
[222]	Whalley et al.	2004	Aberdeen participants (64 years old)	Fish oil supplement	Fish-oil-supplement use and erythrocyte n-3 PUFA content associated with better cognitive aging
[223]	Morris et al.	2003	Chicago Health and Aging Project participants (73 years old)	Dietary habits	Total intake of n-3 PUFAs (and DHA) associated with reduced risk of Alzheimer’s disease
[224]	Barberger-Gateau et al.	2007	Three cities cohort participants (75.9 years old)	Mediterranean diet for 4 years	Weekly consumption of fish or regular use of n-3 PUFA rich oils associated to a reduced risk of Alzheimer’s disease
[225]	Devore et al.	2009	Rotterdam study cohort participants (>55 years old)	Moderate fish consumption	Moderate fish consumption not associated to dementia risk
[226]	Kalmijn et al.	2004	Doetinchem cohort participants (45–70 years old)	Dietary habits	Marine n-3 PUFA (fatty fish consumption) inversely related to the risk of impaired overall cognitive function and speed
[227]	Gonzalez et al.	2010	Elderly population of Asturias (75 years old)	Dietary habits	EPA and DHA intake (fish intake) negatively associated with cognitive impairment
[229]	McNamara et al.	2018	Cincinnati participants (62–80 years old)	Supplementation with 1.6 g/d EPA + 0.8 g/d DHA for 24 weeks	Supplementation associated with reduced cognitive symptoms in everyday activities
[230]	Ferrucci et al.	2006	Chianti participants (20–98 years old)	Dietary habits	Plasma n-3 PUFAs associated with lower levels of pro-inflammatory markers (IL-6, IL-1ra, TNF-α, CRP) and higher levels of anti-inflammatory markers (soluble IL-6r, IL-10, TGF-β)
[231]	Alfano et al.	2012	Health, Eating, Activity, and Lifestyle cohort participants (>29 years old)	Dietary habits	Higher intake of n-3 PUFAs associated with decreased inflammation (CRP level) and decreased aspects of fatigue
[232]	Farzaneh-Far et al.	2009	Heart and soul cohort participants (>64 years old)	Dietary habits	Inverse association between red blood cell n-3 PUFA levels and the inflammatory markers CRP and IL-6

CRP: C-reactive protein; DHA: docosahexaenoic acid; EPA: eicosapentaenoic acid; PIVUS: Prospective Investigation of the Vasculature in Uppsala Seniors; PUFA: polyunsaturated fatty acids.

**Table 5:** Evidence supporting a role of dietary n-3 PUFAs during aging in animals.

Ref.	Authors	Year	Animals	Treatment	Key Findings
[30]	Labrousse et al.	2012	20-month-old C57BL/6J	Supplementation in EPA and DHA from 20 to 22 months (25 mg and 15 mg/d)	n-3 PUFA supplementation reduces hippocampal cytokine expression and astrocyte morphology and restores spatial memory deficits
[233]	Gamoh et al.	2001	100-week-old Wistar rats	Supplementation in DHA (300 mg/kg/d) for 5 weeks	n-3 PUFA supplementation decreases the number of reference memory errors and working memory errors
[234]	Petursdottir et al.	2008	10-month-old SAMP8 mice	Supplementation in EPA and DHA for 8 weeks (11.7% EPA and 14.3% DHA in the diet)	n-3 PUFA supplementation delays cognitive decline through n-3 PUFA incorporation into brain phospholipids
[235]	Bhattacharya et al.	2007	6-week-old C57BL/6 mice	Supplementation with EPA and DHA (400–600 mg/d) for 8 weeks	n-3 PUFA supplementation decreases pro-inflammatory cytokine production (IL-6, IL-1β, TNF-α) in peritoneal macrophages
[236]	Jia et al.	2006	7-week-old B6C3F1 mice	Supplementation in EPA and DHA for 4 weeks (35 mg and 150 mg/d)	n-3 PUFA supplementation suppresses IL-6 transcription in macrophages in a model of nephropathy
[237]	Yaqoob and Calder	1995	High fat diet MF1 mice	Supplementation in EPA and DHA (120 mg and 50 mg/d) for 8 weeks	n-3 PUFA supplementation decreases macrophage TNF-α and IL-6 production
[238]	Sadeghi et al.	1999	Adult C57Bl/6 under high fat diet (20%)	Supplementation in EPA and DHA for 5 weeks (100 mg and 100mg/d)	n-3 PUFA supplementation decreases plasmatic TNF-α, IL-6, and IL-1β concentrations after LPS injection
[239]	Vreden et al.	1995	5-week-old Brown Norway rats	Supplementation with 14% fish oil for 6 weeks	n-3 PUFA supplementation reduces IL-1β production in macrophages
[240]	Miguelez et al.	2006	Adult Sprague-Dawley rats	Supplementation in EPA and DHA for 6 weeks	n-3 supplementation decreases plasma IL-6 levels following an acute challenging dose of exogenous human IL-1β
[241]	Minogue et al.	2007	22-month-old Wistar rats	Supplementation in EPA for 4 weeks (125mg/d)	EPA supplementation attenuates IL-1β and IFN-γ concentrations and reduces JNK expression in hippocampus, associated to a reduction in age- and Aβ-induced deficits in LTP

DHA: docosahexaenoic acid; EPA: eicosapentaenoic acid; LTP: long term potentiation; PUFA: polyunsaturated fatty acids.
